# Specialist antenatal clinics for women at high risk of preterm birth: a systematic review of qualitative and quantitative research

**DOI:** 10.1186/s12884-017-1232-9

**Published:** 2017-02-02

**Authors:** Reem Malouf, Maggie Redshaw

**Affiliations:** Policy Research Unit in Maternal Health and Care, National Perinatal Epidemiology Unit, Nuffield Department of Population Health, University of Oxford, Old Road Campus, Headington, Oxford, OX3 7LF UK

**Keywords:** High risk pregnancy, Preterm birth, Systematic review, Preterm birth clinic

## Abstract

**Background:**

Preterm birth (PTB) is the leading cause of perinatal morbidity and mortality. Women with previous prenatal loss are at higher risk of preterm birth. A specialist antenatal clinic is considered as one approach to improve maternity and pregnancy outcomes.

**Methods:**

A systematic review of quantitative, qualitative and mixed method studies conducted on women at high risk of preterm birth (PTB). The review primary outcomes were to report on the specialist antenatal clinics effect in preventing or reducing preterm birth, perinatal mortality and morbidity and women’s perceptions and experiences of a specialist clinic whether compared or not compared with standard antenatal care. Other secondary maternal, infant and economic outcomes were also determined. A comprehensive search strategy was carried out in English within electronic databases as far back as 1980. The reviewers selected studies, assessed the quality, and extracted data independently. Results were summarized and tabulated.

**Results:**

Eleven studies fully met the review inclusion criteria, ten were quantitative design studies and only one was a qualitative design study. No mixed method design study was included in the review. All were published after 1989, seven were conducted in the USA and four in the UK. Results from five good to low quality randomised controlled trials (RCTs), all conducted before 1990, did not illustrate the efficacy of the clinic in reducing preterm birth. Whereas results from more recent low quality cohort studies showed some positive neonatal outcomes. Themes from one good quality qualitative study reflected on the emotional and psychological need to reduce anxiety and stress of women referred to such a clinic. Women expressed their negative emotional responses at being labelled as high risk and positive responses to being assessed and treated in the clinic. Women also reported that their partners were struggling to cope emotionally.

**Conclusions:**

Findings from this review were mixed. Evidence from cohort studies indicated a specialist clinic may be a means of predicting or preventing preterm birth. Testing this in a randomised controlled trial is desirable, though may be hard to achieve due to the growing focus of such clinics on managing women at high risk of preterm birth. Ongoing research has to recognize women’s experiences and perceptions of such a clinic. Further clarification of the optimal referral route and a clear and standardized management and cost economic evaluation plan are also required. Fathers support and experience of PTB clinics should also be included in further research.

## Background

An estimated 15 million babies are born prematurely (<37 weeks’ gestation) each year and approximately one million die annually due to complications of prematurity [1]. The rate of preterm birth ranges from 5% in some European countries to 18% in some African countries [1]. In 2012, the national preterm birth rate in England and Wales was estimated to be 7% of all births [2]. Preterm birth remains the main cause of perinatal morbidity and mortality worldwide [3], it is the second leading cause of death in children under 5 years of age and the single most important direct cause of death in the first month of life [4]. The complications of preterm birth arise from immaturity in organ developments and survivors could suffer from long term disabilities. Therefore, a minor reduction in preterm births would lead to a substantial cost reduction [5]. Despite the improvement in neonatal care in recent decades and the marked impact on both mortality and morbidity, the incidence of preterm birth is still rising [6, 7]. A high proportion of preterm multiple gestations associated with assisted reproductive technologies is also an important contributor to the overall increase in preterm births. Singleton pregnancies after in-vitro fertilisation are also at increased risk of preterm birth [8].

The implications of preterm birth are not only associated with a significant neonatal hospital cost [9] but also with emotional and economic costs for the family and society [10]. Many pathways can lead to preterm birth (PTB) some resulting from pregnancy complications and others precipitated by concern for the health of the mother or the baby. However, spontaneous labour is responsible for 70–80% of preterm births and 20 to 30% occur as a result of intervention for maternal or foetal problems [11]. Factors contributing to an increased risk of spontaneous labour have been identified: prior preterm birth, Black ethnicity, advanced maternal age, lower and higher BMI, lower socioeconomic status, cervical injury or previous surgery and multiple pregnancy [12–14].

Various preventive options and tests are currently in use to prolong pregnancy such as progesterone supplementation, treating intra-uterine infection, surgical closure of the cervix with cerclage, improvement in maternal nutrition and lifestyle modification [15]. However, the complexity of managing these pregnancies led to the establishment of specialist preterm prevention clinics. Many hospitals have specialist gynaecology clinics, but relatively few have specifically preterm birth clinics, whose fundamental aim is to assist in avoiding preterm birth and reduce the associated perinatal mortality and morbidity [14]. The clinics focus on reducing preterm birth by providing a package of specialist care for high-risk women that could involve serial sonographic assessment, foetal fibronectin testing, vaginal PH testing and other management to prevent early labour. A Cochrane review [16] concluded that there was no clear evidence that specialized antenatal clinics reduce the preterm birth rate, reviewing only three randomised controlled trials from the USA. The three included studies were conducted in the 1980s, when many screening tests and ultrasounds, such as assessment of the cervix length and foetal fibronectin test that are currently in use in the clinic were not available. Moreover, the interventions across the studies were generally similar, offering only education about signs and symptoms of preterm birth in addition to more frequent antenatal visits to high risk women. The outcomes of interest across the studies were preterm birth rate and gestational age at delivery, with no reporting on maternal health and long term infant outcomes. Thus we believe it is necessary in our review to bring together evidence from primary quantitative and qualitative research to evaluate such clinics further.

### Objectives

The review objective is to comprehensively assess the efficacy of specialist preterm clinics in preventing preterm birth and to report on the women’s perceptions and experiences of accessing such services.

## Methods

A review protocol was published at PROSPERO with a registration number CRD42015026976 and this is available at http://www.crd.york.ac.uk/PROSPERO/display_record.asp?ID=CRD42015026976.

In conducting this review, we followed the standard Preferred Reporting Items for Systematic Reviews and Meta-Analyses (PRISMA) checklist [17].

### Types of study

Although randomised controlled trials (RCTs) provide the best evidence for estimating the effectiveness of any health interventions [18], this type of clinic is an accepted part of primary antenatal care in many settings and conducting RCTs may not be ethically possible. Evidence from both qualitative and quantitative research are therefore considered for inclusion in this review. All quantitative research methods, including randomised controlled trials, cohort studies, case-controlled studies, time series studies, cross-sectional and pre-post evaluation studies. Any observations and questionnaires which produce quantitative results were sought for inclusion. Qualitative research include range of designs: interviews, participant and non-participant observation, focus groups and documentary analyses. Studies with mixed method designs were considered eligible for inclusion.

### Types of participant

Studies conducted on women at high risk of preterm labour were eligible for inclusion in this review. Studies enrolling pregnant women with a singleton or multiple pregnancies were included.

### Types of intervention

Specialist preterm prevention clinic: this could be called a specialist antenatal clinic, preterm birth prevention clinic, multi-disciplinary antenatal clinic and miscarriage follow-up clinic (this list is not exhaustive) compared or not compared with standard antenatal care. Studies involving other specialist antenatal clinics such as diabetes, hypertension and twins clinics were excluded.

### Types of outcome measure

The primary outcomes relate to preterm birth defined as birth less than 37 completed weeks’ gestation, very preterm birth (<34 weeks’ gestation), moderate prematurity (32–33 weeks), severe prematurity (28–31 weeks) and extreme prematurity (<28 weeks), perinatal mortality and morbidity (neonatal intensive care admission, respiratory distress syndrome and disability in early life) and measures reflecting women’s satisfaction and wellbeing. Other outcomes such as delivery mode, birth weight and cost associated with running the clinic (number or antenatal visits, hospital admission and length of maternal and neonatal hospital stay) were all considered.

### Search and screening strategy

We developed a sensitive search strategy for five databases: MEDLINE, PsycINFO, Embase, Cinahl, and Cochrane. The strategy was designed to search the title and abstract fields or the thesaurus terms for pregnancy, antenatal, prenatal, prepartum, or preterm adjacent to following truncated words: project, program, service, clinic, meeting, or class. This set was then combined with the terms for high risk pregnancy such as hypertension, eclampsia, diabetes, HIV, epilepsy, previous preterm, or placenta praevia. We did not apply a qualitative search filter, and the “qualitative” term was introduced as the indexing system of databases only since 2003. We limited the search to English language references published from 1980 to March 2015. (See Appendix A for MEDLINE search report).

All retrieved references were imported into a referencing software program (ENDNOTE version 7). Two reviewers independently assessed the studies for inclusion in the review and any disagreement was resolved through discussion. Conference proceedings, reviews reference lists were also hand searched to identify additional studies.

### Methodological quality assessments

The risk of bias of studies of a quantitative type were assessed by applying the Cochrane Effective Practice and Organization of Care group (EPOC) [19] criteria. The tool assesses the risk of bias for the following domains: sequence generation, allocation concealment, blinding, incomplete outcome data, selective reporting, baseline characteristics, baseline outcomes, protection against contamination and other bias. Each domain was given one of the following ratings: “yes”, “no” or “unclear”.

The Critical Appraisal Skills Programme (CASP) [20] for evaluating the risk of bias of studies of qualitative design was implemented. This tool has a checklist of ten questions covering the study objectives and rationale, study methods, study design, study value, recruitment strategies, method of data collection, information on ethical approval, researcher-participant relationship, reliability and validity method of analysing data and reporting of findings. Each domain was given “yes”, “no” or “unclear”.

The quality assessment was conducted independently by the two reviewers and any discrepancies in quality rating were resolved by discussion. For low risk of bias studies the low risk should be given to all domains in the risk of bias tool; for medium risk of bias studies at least 1 of the risk-of-bias criteria was not met, and a high risk of bias studies was assigned to studies with two or more risk-of-bias domains of the risk of bias tool. Unclear risk of bias was assigned for the studies when risk-of-bias criteria was poorly reported.

#### Data collection and analysis

Individual data extraction forms were designed for the quantitative and qualitative studies. The form for quantitative studies holds information about the study design, participants’ characteristics, components of care provided in the clinics, outcome variables and reported results. For qualitative studies the study setting, study aims, ethics, participants’ characteristics, and recruitment and sampling methods, methods used for data collection and analysis, reported themes and study conclusion were extracted.

Studies were summarized and grouped by their study designs and sub-grouped by their reported outcomes. A narrative synthesis only was implemented for data extracted from quantitative studies, as we identified heterogeneity and variation across the included studies. The heterogeneity arose from different study designs, variation in study inclusion criteria, intervention and reported outcomes. We originally planned to undertaken a meta-synthesis of data extracted from qualitative studies, however we only reported the common themes from one qualitative study found eligible for inclusion in this review.

All data were extracted and cross checked independently by the two reviewers.

## Results

### Results of database searching

**Fig. 1:**
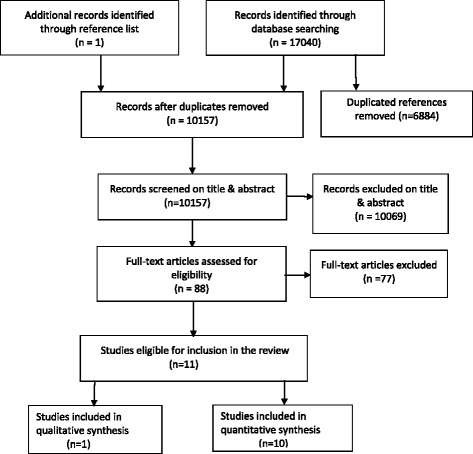
PRISMA flow chart for study selection

The search strategy yielded 10,704 citations all generated from searching data bases electronically. Of these 6884 were duplications and 10,157 were unique study references. We identified 88 relevant references and full texts were retrieved and examined. Seventy-seven studies were excluded and 11 studies met the review inclusion criteria (See Fig. 1). One study was found via checking reference lists of the included studies [21]. The reference list of excluded publications with reasons is available on request from the authors.

### Description of included studies

The review studies were organised by methodological design. Eleven studies met the review inclusion criteria, five were randomised controlled trials [21–25] and five were cohort studies [26–30]. Only one qualitative design study was included in this review [31].

#### Description of randomised studies

We included five randomised controlled trials [21–25] (Studies description is shown in Table 1). All were conducted in the USA from 1985 to 1990. One study was only available as an abstract [21]. Four studies were single centre studies [21, 22, 24, 25] and one [25] was a cluster randomised study involving eight clinics, five in the intervention group and three in the control.

**Table 1:** Characteristics of included quantitative studies

Study ID, design, country	Participants	Intervention	Outcomes	Results
Randomised controlled trials (RCTs)				
1. Iams and Johnson [21], single centre, study duration 1983 to 1986 (abstract only), USA	370 high-risk women based on Creasy scoring system were selected from 2829 women attending antenatal clinic. One hundred eighty-two women received routine antenatal care plus preterm birth prevention clinic the intervention and 188 women received routine antenatal care.	Preterm birth prevention clinic group received education about symptoms and signs of labour and the cervix examined at weekly visits between 20 and 36 weeks’ gestation Control group received standard antenatal care.	1.Preterm labour (intervention vs control): 50/182 vs 40/188, *P* = 0.17 2. Preterm birth < 37 weeks (intervention vs control): 24/50 (48%) vs 35/40 (87.5%), *P* = 0.001	No significant difference between the two groups with regards to the incidence of preterm labour. Significant difference between the two groups with regard to preterm birth among women who developed preterm labour.
2. Main et al. [22], single centre, study duration: 3.5 years, USA	367 black women at gestational age > 18 weeks were at high risk of preterm labour based on Creasy et al. [32] scoring criteria. Inclusion criteria: Black women with gestational age < 18 weeks were referred to the nurse specialist in the Preterm Labour Detection Clinic. Intervention group: *N* = 178, maternal age (yr) 23.9 ± 5.5, gravidity 3.7 ± 1.9, parity 1.4 ± 1.2, abortions ≤ 14 weeks 1.0 ± 1.0, abortion > 14 weeks 0.3 ± 0.7, women with previous preterm delivery 38%, gestational age at first visit (wk) 12.5 ± 3.7. Control group: *N* = 198, maternal age (yr) 24.1 ± 5.1, gravidity 3.8 ± 1.9, parity 1.6 ± 1.5, abortions ≤ 14 weeks 0.9 ± 1.1, abortion > 14 weeks 0.3 ± 0.7, women with previous preterm delivery 43%, gestational age at first visit (wk) 12.0 ± 3.3.	Attending a preterm labour detection clinic on a weekly or biweekly basis from 22 weeks’ gestation and cervical assessment by 1 of 3 physicians at each visit. Also education provided by a nurse specialist regarding subtle signs of labour. High risk control: received usual prenatal care.	1.Preterm deliveries (intervention vs control): <28 weeks: 3% vs 3.9%, *p* = 0.42, 32 < 34 weeks: 6.6% vs 6.2%, *p* = 0.51, <36 weeks: 16.7% vs 13.4%, *p* = 0.46, <37 weeks: 23.2% vs 20.7%, *p* = 0.32, 2. Neonatal outcomes: 5-min Apgar <5 4.5% vs 6.1%, *p* = 0.32. Caesarean birth: 23.7% vs 21.2%, *p* = 0.64. NICU admission: 10.4% vs 16.4%, *p* = 0.32. Length of stay > 5 days: 21.4% vs 18.7%, *p* = 0.33. Stillborn: 4.8% vs 2.9%, *p* = 0.53. Neonatal deaths: 0% vs 0.7%, *p* = 0.48. 3. Cost/Hospital charges: Maternal charges: $5687 ± 4222 vs $5846 ± 4872, *p* = 0.97 Neonatal charges: $4958 ± 26,491 vs $4287 ± 24,247, *p* = 0.83. 4. Maternal hospital admission: Mean no. maternal hospital admissions: 1.7 ± 1.1 vs 1.3 ± 0.7, *p* = 0.0001, Women with one or more antepartum admissions: 44% vs 26%, *p* = 0.001.	No significant differences between the two high risk groups with respect to mean gestational age at delivery, birth weight or percentage delivering before term.
3. Mueller-Heubach [23], study duration 3 years between September 1984 and August 1987, USA	5457 women were scored for risk of preterm birth using the Creasy scoring system 1980, and 18.1% were classified as high risk these were randomised into two groups. Exclusion criteria: Patients registered after 28 weeks’ gestation.	The intervention group received weekly cervical examinations and teaching about signs and symptoms of preterm labour. Health care professionals received similar instructions. Historical control was used due to high contamination. The control group received the usual antenatal care.	Preterm birth rate (intervention vs control): 22.1% vs 20.8%, *p* > 0.05 Preterm birth in year one: 13.7%, in year two 9.3%, *p* < 0.001 and in year three 8.9%. Neonatal death (second and third year): 5/1755 vs 11/1203 the incidence: 2.8/1000 vs 9.1/1000.	There was no difference in preterm birth between the intervention and the control. There was a significant reduction in preterm birth rate in year 3 compared to year 1. There was a significant decrease in the neonatal death in the second and third year of the intervention compared with the control.
4. Goldenberg et al. [24], five centres, study duration 1982–1986 (singleton and, multiple pregnancies), USA	1000 high risk women were randomized to intervention or control. Seventy percent were black and 35% were younger than 20 years and 4% were 35 years or older. 3.5% in the intervention had multiple pregnancy and 4.2% in the control. Inclusion criteria: women with an estimated date of delivery between 1 November, 1982 and April 1, 1986, at < 30 weeks gestational age, women were classified as high risk based on a score of 10 or more on the based on Creasy et al. [32] criteria.	The intervention group attended the clinic weekly and pelvic examination and education about preterm signs and symptoms. Primary care was provided by a specially trained nurse who saw the same woman. Women in the control group received usual prenatal care.	1. Pregnancy outcomes (intervention vs control): Spontaneous preterm labour: 26.9% vs 16.3% Spontaneous premature rupture of membranes (PROM) 6.3% vs 4.4% Preterm delivery incidence: 6.3% vs 2.5% Spontaneous delivery < 28 weeks: 2.7% vs 1.3%, *p* > 0.05 Spontaneous delivery <36 weeks: 11.8% vs 10.5%, *p* > 0.05 Spontaneous delivery < 37 weeks: 15.9% vs 14.2%, *p* > 0.05 Birth weight 1500–2499 g: 37.7 ± 3.8 vs 38.1 ± 3.1, *p* > 0.05 Mean birth weight: 2892 ± 771 vs 2935 ± 679, *p* > 0.05 2. Neonatal outcomes: Respiratory distress syndrome: 5.9% vs 3.8%, *p* > 0.05 Hyperbilirubinemia: 7.9% vs 9.4%, *p* > 0.05 Necrotizing enterocolitis: 0.6% vs 1.8%, *p* > 0.05 Patent ductus arteriosus: 2.4% vs 1.6%, *p* > 0.05 Interventricular haemorrhage: 1.8% vs0.4%, *p* < 0.05 Congenital anomaly: 6.75 vs 7.8%, *p* > 0.05 Sepsis: 0.8% vs 0.8%, *p* > 0.5 Hypoglycaemia:2.3% vs 4%, *p* > 0.5 Need for resuscitation: 8.2% vs 8%, *p* > 0.05 NECU: 27.4% vs 26.6%, *p* > 0.05 Time on ventilator: <12 h:93.5 vs 97.4, *P* < 0.05; >12 h: 6.5% vs 2.4%, *p* < 0.05 Babies days in hospital: ≤7: 89% vs 91.8%, *p* > 0.5; >7 11% vs 8,2%, *p* > 0.05	Preterm labour diagnosis and spontaneous preterm PROM diagnosis were higher in the intervention group, but the difference was not significant. No significant difference between the groups on most the neonatal outcomes.
5. Hobel et al. [25], multicentre study, 5 clinics in the intervention and three in the control, recruitment lasted from 1983 to 1986, USA	1774 high-risk women in the intervention clinics and 880 in the control clinics. Women were predominantly Hispanics. Inclusion criteria: Had a gestational age of <31 week, no disabling condition, and were English or Spanish speaking. Exclusion criteria: major congenital anomaly, multiple births, pregnancies with missing charts of cost information.	Intervention group received preterm birth prevention education plus increased antenatal visits to the clinic and selected prophylactic interventions. Visits were scheduled at 2 weeks intervals, 3 educational classes about preterm birth prevention, nutritional and psychosocial screening and offered treatment when it was needed. The control clinics offered visits at 4 weeks intervals up to 30 weeks ‘gestation, then every 2 weeks from 30 to 35 weeks’ gestation, then weekly until delivery.	1. Number of clinic visits(intervention vs control): 6.4 ± 3.4 vs.9 ± 2.5, *p* < 0.05 2. Preterm rate: 7.4% vs 9.1%, *p* = 0.063 3. Birth weight <2500gm: 5.8% vs 6.4%, *p* = 0.15 4. Gestatioanl age: 39.8 ± 2.3 vs 39.9 ± 2.5, *p* = 0.38 4. Inpatient costs per New born: <37 weeks: (*n* = 95, 17,206 ± 3995 vs *n* = 55, 31,129 ± 8572) ≥37 weeks: (*n* = 70, 2025 ± 273 vs *n* = 70, 2763 ± 628) 5. Average new born inpatient cost:$3146 vs $5342	No significant difference between the two groups with regards to the incidence of preterm birth, low birth weight and gestational age. High risk prevention clinics had an average cost savings of $2196 for new born care (*p* = 0.2).
Cohort studies				
1. Herron et al. [26], prospective-cohort, single centre, between July 1, 1978 and June 30, 1979, USA.	Patients were screened based on the Creasy criteria 1980 and divided into two groups: 176 (15.2%) women assigned to the high risk group and 974 (84.8%) to the low-risk group.	For the high risk group: The intervention involved: the first visit to the clinic included education regarding the signs and symptoms of preterm labour and training the participants in self-detection of painless contractions. Weekly antenatal visit to the clinic, if the symptoms of painless labour occurs then patients were monitored for 1–2 h. Reporting to the clinic immediately if one of the preterm signs and symptoms occurred. AT the weekly visit the pelvic examination was performed by the same physician. If preterm labour occurred then patients admitted to hospital and tocolytic therapy was given. Staff training and education to prompt response to patients’ complaints, of any preterm signs and symptoms, early admission to patients having a mild increase uterine activity, aggressive therapeutic approach in patients with documented preterm labour, awareness of long term side effects of the tocolysis.	1.Preterm labour(comparing high risk group to low risk): 30/176 (17.5%) vs 24/974 (2.5%), *p* < 0.05, 2. Preterm delivery (comparing high risk group to low risk group): 7/176 (4%) vs 9/974 (0.9%), p > 0.05. 3. Men gestational age at delivery (comparing high to low group): 33.7 ± 2.6 vs 33.3 ± 3.6 weeks 4. Preterm birth ≤ 36 weeks at year 1 after introducing the clinic: 2.4% compared with 6.75% before the clinic.	A significant decrease in preterm birth with the clinic.
2. Manuck et al. [27], Retrospective cohort, multi-centre study from 17 hospitals, participants’ enrolment from 2008 to 2010, USA.	Inclusion criteria: Single pregnancy, previous PTB <35 weeks. Exclusion criteria: Women who delivered preterm babies <37 weeks due to medical or foetal complications, eg, preeclampsia, foetal growth restriction. Women excluded from the study analysis if they had a history of incompetent cervix (painless cervical dilation <24 week’s gestation). Total number of patients: 223 PTB clinic group: *n* = 70 Maternal age 28.5 years, white 83.1%, smoking 3.4%, married 86.4%, primary obstetrics provider is perintalogist 18.6%; number of PTB <37 weeks 1.7 (mean) Usual care group: *n* = 153 Maternal age 28.8% years, white 88.8%, smoking 9.8%, married 83%, primary obstetrics provider is perintalogist 11.8%; number of PTB <37 weeks 1.6 (mean)	The recurrent PTB prevention clinic includes three visits (10–18 weeks, 19–24 weeks, and 28–32 weeks): Detailed obstetric history and personal recurrence risk assessment: at visit 1 (10–18 weeks) Screen for BV and treat if positive with oral metronidazole at all three visits. Urinalysis : at all three visits Urine culture: at all visits (symptoms positive or urinalysis is positive). Transvaginal cervical length: at all visits. Cervical length <2.5 cm is abnormal. Offer 17 alpha hyroxyprogesterone caproate: at visit one for all patients, patients who declined were offered the treatment again at week 24 if cervical shortening is noted. Usual care group: Managed by their primary obstetrician without being referred to the clinic.	Primary outcome (PTB clinic vs usual care): 1. PTB < 37 weeks,%: 48.6% vs 63.4%, *p*=0.02 2. PTB < 37 weeks,%: 5.7% vs 13.7%, *p*=0.08 3. Delivery GA, mean wk: 36.1 vs 34.9, *p* = 0.02 Secondary outcomes: 1. Neonatal morbidity, %: 5.7 vs 16.3, *p* = 0.03 2. NICU admission, %: 44.3 vs 41.2, p = 0.66 3. Mean inpatient maternal cost: $6929 vs $7706, p = 0.48 4. Mean inpatient neonatal cost: $11,818 vs $15,662, *p* = 0.05	28% reduction in the risk of recurrent PTB <37 weeks and >1 week of pregnancy prolongation and reduced the rate of major neonatal morbidity with the intervention.
3. Karkhanis et al. [28], retrospective-cohort from November 2007 to January 2009, Birmingham-UK (abstract)	180 high risk women, mean age 29.85 years (18–41), mean BMI = 27.52 kg/m2, *N* = 158 with previous preterm labour or mid trimester loss.	All patients in the preterm prevention clinic underwent serial transvaginal scan monitoring and infection screening between 16 and 28 weeks. 40 women underwent cervical cerclage and progesterone 35 women received progesterone only	1. Term delivery > 37 weeks: *n* = 123/180 2. Term delivery >37 weeks after one preterm delivery (PTD): 79% 3. Term delivery > 37 weeks after 2 PTD:71% 4. Term delivery >37 weeks after 3 PTD:60% 5. NICU admission: *n* = 36 babies 6. Infant mortality: *n* = 7	The preterm prevention clinic reduced prematurity rate.
4. Burul et al. [29], retrospective-cohort, clinic cases from January 2005 to December 2008, London-UK (abstract).	210 cerclage cases: 85 cases before the establish of the clinic and 125 afterwards	Cervical cerclage	1. Elective cervical cerclage 44% before the clinic vs 88% after establishing the clinic 2. GA at delivery 28 + 2/40 before the PTBC compared with 35 + 2/40 with the clinic care	
5. Cohen et al. [30], audit of two London preterm surveillance clinics between January 2013 and May 2014, UK (abstract).	509 pregnancies reviewed; mean age 33.6 years (18–49 years), BMI 24.4 (range 17–48), 59% White and 15% Afro-Caribbean. Reasons for referral to the clinics: Previous cervical treatment (50%) Previous preterm birth before 34 weeks 926%), mid trimester miscarriage (MTL) (17%) Uterine anomalies (2%) Multiple pregnancy (3%)	Clinic interventions: Cervical shortening found in 44% Progesterone supplementations 25% Cervical cerclage 27%	Preterm delivery: <28 weeks 0.7% delivered <34 weeks 4% delivered <37 weeks 11% derived	Early referral to the clinics for better monitoring.

Overall, 8986 women were involved and 5796 were categorised as being at high risk of preterm birth using the Creasy et al. [32] scoring system (low risk < 10 or high risk >10) in four studies [21–24], and one with a specifically designed risk assessment tool [25]. Similar entry criteria were utilized across the studies with women at less than 30–31 weeks gestational age at clinic first visit with no major congenital anomalies or disabling conditions included. Multiple pregnancy was an exclusion criteria in one study only [25]. The demographic characteristics and factors increasing the risk of preterm were not distinctly different, in the three studies [22, 24, 25] which were carried out in predominately Black or Hispanic women (See Table 1 for details). The intervention differed slightly between studies. In Iams and Johnson [21] and Muller-Heubach [23], women in the intervention group received a weekly visit to the clinic between 20 and 36 weeks gestation in which signs and symptoms of preterm labour were taught and the cervix was examined. Healthcare providers in Muller-Heubach [23] changed the study design by offering the intervention to all participants, and a historical control group was thus established. Women were also seen weekly or biweekly starting at 22 weeks’ gestation and offered a comprehensive education in Main et al. [22]. Whereas, in Hobel et al. [25], women attending five clinics received the intervention, and in three the control. High risk women in the intervention cluster received three educational classes on preterm birth prevention and visits to the clinic scheduled every 2 weeks. In a nested study, women were also randomised to one of the four following interventions: protocols of bed rest, psychosocial support, Provera (progesterone) or placebo, or no additional intervention. Additionally, nutritional screening, psychosocial support and crisis intervention were offered to participants from both groups. In all studies the women in the control groups were assigned to receive the usual antenatal care. Ultimately, all five studies had similar primary outcomes of preterm labour and gestational age at delivery.

#### Description of cohort studies

Five cohort studies [26–30] were included in this review (Table 1 is a summary of the study characteristics). Three studies were conducted in the UK [28–30] and two in the USA [26, 27]. Herron et al. [26] was a prospective cohort single centre study where participants were assigned one of two group, high and low risk, based on the Creasy et al. [32] criteria [30]. Participants were then instructed on how to identify early signs of preterm labour and to be followed weekly in a specialist clinic, in addition to their usual antenatal care. If preterm labour occurred, women were admitted to hospital for further treatment. In Manuck [27], 223 women were identified from a clinic data base retrospectively. Women were included if they had at least one PTB < 35 weeks’ gestation and one subsequent singleton pregnancy carried to at least 20 weeks gestation. Three clinic visits were scheduled at 10–18 weeks, 19–24 weeks and 28–32 weeks gestation. Screening for bacterial vaginosis (BV), urine culture and transvaginal ultrasound for cervix length were performed at each visit. Hydroxyprogesterone was also offered to all women. The study primary outcome was recurrent PTB < 35 weeks’ gestation.

The three most recent UK studies were published only as abstracts [28–30] and involved a retrospective case-note analysis of patients registered at the clinics. In Burul [29] the focus of the study was to collect data on cervical cerclage and pregnancy outcomes. A total 210 cerclage cases were identified at the PTB clinic, 85 cases before the PTB clinic was established (January 2005–December 2012) and 120 cases since January 2005–December 2012.

Karkhanis et al. [28] reviewed the clinic notes of 180 women from November 2007 to November 2009. All women underwent serial transvaginal scans and infection screening between 16 and 28 weeks. Forty women underwent cervical cerclage and 35 received progesterone.

An audit of two London preterm surveillance clinics between January 2013 and May 2014, described by Cohen et al. [30] aimed to assess the outcomes of 509 high risk pregnancies, among which 27% of women underwent cervical cerclage and 25% received progesterone.

#### Description of qualitative studies

One qualitative study [31] conducted in a single centre in the North West of England was included in this review (Table 2 is a summary of the study characteristics). Data were collected by a mixture of focus groups and one to one interviews. Fourteen Women with high risk pregnancies and at risk of preterm birth who were referred to a specialist antenatal clinic for their antenatal care were interviewed. Three focus groups (*n* = 4), (*n* = 2), (*n* = 4) and 4 individual interviews were conducted. Interviews took place in the clinic or the women’s homes. Data on gravidity, parity, current treatment and demographic data were collected prior to interview. Women were encouraged to discuss their views of high risk pregnancy and their individual care and their management which could include activity restriction, inpatient admission, antibiotics, aspirin and progesterone treatment. Data were analysed thematically.

**Table 2:** Characteristics of included qualitative study

Study ID, country	O’Brien et al. [31], UK
Study Aims	High risk pregnant women’s views on attending a specialised antenatal clinic.
Ethics	Study was reviewed by the hospital’s Research & development committee and gained ethical approval from local research ethics committee.
Participants	Women who had a previous preterm birth, experience antenatal care for the current pregnancy was provided in preterm clinic and English speaking. Women were excluded if they had a known foetal malformation.
Recruitment	Specialist preterm clinic.
Sampling method	Women were identified for inclusion in the study through obstetrician referral.
Participants characteristics	37 women were interested in participating in the study and 14 were interviewed. Age range 23–44 years; 13 were white and one Black Caribbean. Gestational age an interview range (14–32 weeks).
Data quality rating	Two independent researchers analysed the data.
Data collection	Three focus groups and face to face interviews.
Data analysis	Interpretative approach (thematic coding method) was used.
Data extracts	Data transcribed anonymously, coding and categories and themes were developed by two researchers.
Themes	1. Balancing the risks: Women were aware of their risk, but viewed positively due to the extra care (“I would prefer to know and I would see it as a positive thing because you would expect that they would monitor you closely and if necessary give you medication or obviously try and lower the risk somehow to have a successful pregnancy”). 2. Threat of preterm labour: All women felt paranoid about potential signs or symptoms of PTB “Just get through this bit. 3) Personal coping buy developing strategies to survive the pregnancy however, women tried not to focus on their pregnancy avoiding bonding with the baby and were reluctant to look too far to the future. a) Recognizing that something does not feel right: Ignoring the warning signs of PTL with previous pregnancies, however, the PTB was realised they were feeling guilty and not ignoring their intuition again: (“When I look back, leading up to actually having her there were some little signs. And I was very much ignoring them because I was thinking I was being paranoid and silly… the promise that we made to ourselves and particularly to myself was that I am just not going to take any risks….. I don’t care if anyone thinks I ‘m paranoid, you know, or nuts, whatever, as long as I eventually have a healthy baby”). Some women struggled with the health professional to have their concerns taken seriously. Some felt worse after interactions with health professionals in the clinic. c) Need regular reassurance from health professionals were not always sensitive to women’s worries about the risk of PTL, felt better with the routine reassurance of the clinic screening and scanning.

#### Quality assessment of quantitative research

The quality of studies included in this review was mixed, varying from good to low. Two of the included five randomised trials [24, 25] were considered good quality; the other two RCTs [22, 23] were low quality and one RCT [21] was published as an abstract and information to assess the study quality were missing. All four cohort studies were considered low quality (See Table 3 for details).

**Table 3:** Risk of bias of quantitative studies based on the EPOC tool

Study/year	Selection bias	Allocation to intervention	Performance bias	Baseline differences in characteristics	Baseline differences in outcomes	Contamination	Attrition bias	Selective reporting	Other bias
Randomized controlled trials (RCTs)									
1. Hobel et al. [25]	Low: Cluster randomization with a restricted block	Low: cluster randomization.	Low: women were not aware of their intervention status nor the clinics teams	Low: participants were comparable with respect to age, marital status, gravidity, parity and preterm birth rate	Low: no difference between the groups with regards to high risk preterm problems at baseline	Low: intervention was provided on a clinic-basis rather than patients	Unclear: the number of women who left the study was not reported	Low	low
2. Main et al. [22]	High: a random numbers table was used for the first 479 participants then the second sample of464 women was divided into groups by birthday date	High: women’s’ date of birth was used to allocate women to intervention or control	Low: In the control group neither the doctors nor the women were made aware they were at high risk of preterm birth.	Low: no differences between groups with respect to maternal age, gravidity, parity, previous preterm deliveries, and gestational age at first visit	High: More women with previous preterm birth were assigned to the intervention.	High: 8 women from the control group transferred to the clinic	High: insufficient reporting on the rate of attrition.	Unclear	Low
3. Iams and Johnson [21]	Unclear	Unclear	Unclear	Unclear	Unclear	Unclear	Unclear	Unclear	Unclear
4. Goldenberg et al. [24]	Low: randomization by a randomization officer	Unclear: allocation to intervention or control method was not reported	Low: nobody was aware of the intervention status	Low: No significant difference between high-risk group and high-risk control with regard to number of birth, race, age and parity	Low: no differences of previous preterm birth and multiple pregnancies between the two groups	low	Unclear: the number of missing women was not reported	Low	Low
5. Mueller-Heubach [23]	Low: participants were selected to intervention and controlled randomly	Unclear: method of allocation to intervention or control was not reported	High: nurses were aware of the intervention status of the participants	Low	Low	High: a historical control was used in the analysis	Unclear	low	low
Cohort studies									
1. Herron et al. [26]	High	High	High	Low	Unclear	Low	Low	Low	Low
2. Manuck et al. [27]	High	High	High	Low	Low	Low	Low	Low	Low
3. Karkhanis et al. [28] (abstract)	High	High	High	Unclear	Unclear	Low	Unclear	Unclear	Unclear
4. Burul [29] (abstract)	High	High	High	unclear	Unclear	Low	Unclear	Unclear	Unclear
5. Cohen et al. [30] (abstract)	High	High	High	Unclear	Unclear	Low	Unclear	Unclear	Unclear

For the risk of bias assessment in the randomised studies, the allocation concealment technique was described in the three studies [22, 24, 25]. Hobel et al. [25] was a cluster randomisation study with eight clinics allocated to intervention and control using the blocked technique. A quasi-randomisation method was used in Main et al. [22] whereas, in Iams et al. [21] and Mueller-Heubach [23], little was available on study methodology and randomisation allocation was reported with no further information. For this type of intervention blinding of women and health care professionals is difficult as both would be aware of the frequent visits to the clinics. A cluster randomisation method at the clinic level would be the preferable approach to reduce bias associated with contamination between the intervention and control groups. In three RCTs [22, 24, 25] women were not aware of their intervention status. In both Main et al. [22] and Goldenberg [24] health care professionals were not aware of the intervention status. In Mueller-Heubach [23], a high contamination occurred between the intervention and the control, resulting in the use of historical controls. The numbers of participants lost to follow-up for most outcomes were not reported clearly in most studies [22, 24, 25].

All five cohort studies were rated at high risk of bias. Three studies were published as abstracts and information on their methodology was absent. Only one study was a prospective cohort [26] and involved a good sample size (*n* = 179) in the high risk group and (*n* = 974) in the control. Both were selected from the same clinic. The follow-up rate was sufficient, with only three women missing from year 2 results. The second fully-published paper Manuck et al. [27] was a retrospective cohort study and participants were selected from the same clinic with no baseline difference with regards to maternal age, gravidity, parity and the number of previous preterm births. Potential confounding variables were measured and adjusted for in this study (progesterone prophylactic use, history of spontaneous PTB <28 weeks, maternal smoking, male foetus, a short cervix or carrying of private health insurance). In all cohort studies there is no information about whether outcomes were assessed blindly. The risk of bias from allocation to interventions was high and risk of contamination bias was low across included cohort studies.

#### Quality of evidence of qualitative study

Based on the CASP 2013 criteria O’Brien et al. [31], was a good quality study as reflected in the adequate formulation of the study aims and the appropriate use of qualitative methods (See details in Table 4). The characteristics and the recruitment criteria of the study sample were appropriately specified. Validity of data collection was also established with two different methods for gathering data, focus groups and face to face interviews. Additionally, the reliability of data analysis was established as coding and thematic analysis were conducted by two researchers independently.

**Table 4:** Risk of bias qualitative studies using the CASP tool for qualitative studies

Study ID	O’Brien et al. [31]
Study objective	Yes, understanding the women’s experiences of attending and being referred to the specialist antenatal clinic.
Appropriate method	Yes, qualitative methodology is appropriate to seek women’s experience of the clinic.
Study design	Yes, through focus groups and in depth face to face interviews.
Recruitment strategy	Yes, women were enrolled from a specialist clinic which is a major referral centre in the North West England.
Data collection	Yes, data collected through focus groups and face to face interview. All were recorded and transcribed and data saturation was discussed.
Researcher-participant relationship	Unclear, no information was given.
Ethical approval	Yes, study reviewed by Hospital’s Research and development Committee.
Data analysis	Yes, data was analysed by two independent researchers using the constant comparative method.
Study findings	Yes, three themes were explicitly defined and the credibility of the findings was also clearly discussed.
Study values	Yes, researchers identify a new area for further research.

### Effects of the interventions from quantitative research

The following primary outcomes were addressed across the included studies:

- *Preterm birth: (birth < 37 weeks’ gestation):*
  Results from all RCTs [21–25] showed no significant difference between the intervention and the control groups in preterm delivery (7.4% vs 9.1%, *p* = 0.063; 23.2% vs 20.7%, *p* = 0.32; 22.1% vs 20.8%, *p* > 0.05; 15.9% vs 14.2%, *P* > 0.05; and 22.1% vs 20.8%, *P* > 0.05?) respectively. In contrast, results from cohort studies showed a reduction of preterm birth incidence after the clinic was introduced. A 28% reduction in the risk of preterm birth in comparison to data from women receiving usual care was reported in Herron et al. [26]. In Karkhanis et al. [28], the prematurity rate was reduced and the term delivery > 37 weeks’ figures were reported for women with one (74%), two (42%) and three (41%) previous preterm deliveries.
- *Very preterm birth (birth before 34 weeks’ gestation) and extremely preterm birth (birth <28 weeks’ gestation):*
  Data from two RCTs [21, 24] contributed to both outcomes. There was no significant differences between the number of women attending the specialist clinic and delivering very or extremely preterm babies compared to those receiving usual care.
- *Gestational age at birth:*
  Results from one study [25] showed no significant differences between women attending the specialist clinic mean gestation 39.8 (2.3) and women receiving usual care 39.9 (2.3) weeks, *p* = 0.32. The median gestational age at delivery increased from 28 + 2/40 to 35 + 2/40, *P* = 0.6, in the cohort study reported by Burul et al. [29].
- *Stillbirth:*
  One RCT [22], reported no significant difference between the women in the two groups, with seven deaths reported in the intervention group compared with six in the control.

#### Secondary outcomes

For neonatal outcomes such as birth weight, admission to neonatal intensive care and length of hospital stay, there were no significant differences between women receiving the intervention in comparison to women in the control groups. The only significant difference was more women were treated with tocolytics in the intervention group (*p* = 0.3) in Main et al. [22].

The cerclage rate per 1000 women delivered fell from 6 to 5 as reported by Burul et al. [29], and the gestational age at cerclage placement fell after introducing the clinic (17 + 0/40, 13 + 2–23 + 3 to 15 + 2/40, 12 + 2–23 + 4 weeks, *P* > 0.05). The proportion of rescue cerclage also fell (26% to 12%, *P* > 0.05), whereas the proportion of elective cerclage doubled significantly (44 to 88%).

#### Cost effective outcomes

Three included studies [22, 25, 27] calculated maternal and neonatal cost-effectiveness associated with care in the clinic. In Ross et al. [33], a cost effectiveness evaluation for Hobel et al﻿ [25], data on costs were only available for a sub-group of women and cost were collected for prenatal care, maternal inpatient costs for preterm labour, delivery and postpartum care, and newborn inpatient care cost. The results indicated a net savings of $1768 for every high risk mother-infant pair. The estimated outpatient cost per patient was significantly higher for women attending preterm clinic in Main et al. [22]. Both inpatient maternal and neonatal care costs were higher among women receiving routine care in Manuck et al. [27] as the outpatient cost was not available.

### Findings from qualitative research

In O’Brien et al. [31], women’s response to high preterm risk pregnancy was a mixture of being reassured by the treatments and frequent clinic appointments and feeling anxious and emotionally drained. Therefore, women in this study developed coping strategies during their pregnancy and the following three main themes were emerged: balancing the risks associated with the threat of preterm birth, developing personal coping strategies to survive the pregnancy (focusing on the present and not looking too far into the future) and developing a family coping strategy.

Women also acknowledged that their physical and emotional needs were considered and addressed in the clinic, however their partners who were struggling to cope emotionally were ignored.

## Discussion

### Summary of main findings

A strength of this review arises from searching for evidence from both quantitative and qualitative research studies although those included were predominantly of a quantitative design. This is because our aim was initially to enhance the integrity of review findings, reflecting on women’s perspectives in addition to clinical outcomes.

The review findings were mixed. Evidence from randomised controlled studies suggested that there was no differences between usual care and care provided at a specialist preterm clinic. In contrast, evidence from cohort studies emphasized that a specialist clinic for managing high risk women is associated with a reduction in preterm birth and lower rates of adverse neonatal outcomes. Moreover, results from individual studies sometimes produced mixed results. In Goldenberg et al. [24] results were not in favour of the clinic and some outcome measures such as foetal and neonatal mortality were slightly worse in the intervention group than in the control. This was explained by the poor compliance with the individual clinic visits. The included RCTs in this review were conducted in late 1980s and 1990s before the usage of cerclage or other new management to prevent preterm birth and before the availability of new screening tests such as the foetal fibronectin screening test (fFN). The intervention itself in these old studies was only by increasing the frequency of antenatal visit to weekly or biweekly and educating the pregnant women about preterm labour signs and symptoms.

The included studies referred to specialist clinics which were established to prevent the onset of preterm labour and facilitate its early identification and treatment. Although these clinics shared a similar goal, the studies varied in their primary outcome focus, target populations, study designs, and specific intervention components. Another common component of the clinic was the initial screening for women at risk, which in the earliest included studies involved using the Creasy et al. [32] scoring system to identify high risk women. However, more recent studies have relied on specific screening tests such as measuring cervical length and fibronectin testing to identify this group of women. In general women are most likely to be referred if they have had a previous preterm birth, late miscarriage, multiple pregnancy or cervical surgery [14, 34].

A particular limitation of the available quantitative studies is the absence of data collection relating to women’s mental health and wellbeing in the context of specialist preterm clinic care in addition to the lack of women’s experiences of care. In the single included qualitative study on women’s views, some themes reflected psychological issues, namely their anxieties and a need for continuous reassurance and support. The experiences of women accessing this clinic was only addressed in the one qualitative study included in this review [31], women felt relieved by being labelled as “high risk” of preterm birth and by being referred to the clinic, which had offered them a sense of reassurance and frequent clinical assessments. However, only a small number of participants from a single centre who could speak English were interviewed. The views of women from ethnic minority backgrounds were not heard. Other qualitative studies of women who experienced preterm labour, unrelated to the use of a specialist preterm birth clinic, for example MacKinnon and McIntyre [35] have explored women’s fear about preterm birth, guilt, feelings of being judged and their sense of personal responsibility in preventing labour. Both parents may be involved in clinic attendance, however no studies of fathers’ experience and support in relation to PTB clinics were found.

Another limitation in this review is the lack of accurate economic costing of the clinics with only three studies reporting on relative cost outcomes and using various measure. Results from two studies suggested a cost saving effect of the clinic when compared with standard care, including only inpatient maternal and neonatal care in the economic model. However, the outpatients care cost was higher in the clinic as suggested by the third study.

We conclude that the current literature suggests some benefit of specialist clinics aimed at preventing preterm labour and delivery, but methodological weakness across these studies indicate caution as the most positive reported outcomes are from retrospective cohort studies. While effective intervention may be possible, some risk factors for preterm birth cannot be changed, for example greater maternal age and a previous history of preterm birth. However, the way in which antenatal care is delivered for this population in terms service organisation and care clearly can be changed. First of all the current screening for the risk of preterm birth has changed and the usage of foetal fibronectin testing (fFN) and cervical ultrasound will identify quite a different risk group to those included in earlier studies where the risk of preterm birth was based on the woman scoic-demographics and a previous history of preterm birth. Additionally, other models of antenatal care to prevent and reduce preterm birth such as midwife led continuity of care has been proposed, this is a comprehensive and specialized antenatal clinic-based care or a shared antenatal midwife-obstetric model of care. Alternative antenatal care models are systematically studied and it has been found to be effective in reducing preterm birth for all pregnant women when compared to standard care [36]. Therefore, in arguing for a population health strategy in preventing preterm birth Heaman et al. [37] emphasized that a comprehensive model in preventing preterm birth should be based on targeting the social and economic environment, the physical environment, personal health practices and individual capacity and coping skills, in addition to healthcare services. Thus while maternity services may include such specialist clinics, it must be held in mind that other factors may be more powerfully influencing preterm birth rates and outcomes.

### Review limitations

The lack of meta-analysis to identify the efficacy of such a clinic in reducing preterm birth is one of the major limitations of this review. We are aware of the result of a meta-analysis of the preterm birth outcome in the Cochrane review [16] illustrating that there is no significant difference between a specialist clinic and standard care for high risk women. As stressed earlier, this was a result of combining three old studies only. However, the most recent data on clinic efficacy were collected from four cohort studies and combining data from different study design is not feasible.

Another limitation was the absence of any measurement of the women’s well-being in the included studies.

### Review agreement and disagreements with other reviews

We are not totally with agreement with the conclusion of the 2011 Cochrane review [16], as positive outcomes about the clinic were suggested by more recent cohort studies. The Cochrane review stated that specialist clinics for preterm birth prevention are not effective in preventing preterm labour. As mentioned previously, this was a result of combining results data of three RCTs conducted before 1994, two of which are included in this review [21, 22].

Another and more recent systematic review with meta-analysis [36], looked at the existing models of antenatal care and their effectiveness in reducing preterm birth. Fifteen randomized controlled trials were included and the risk of preterm birth was significantly lower among pregnant women receiving alternative antenatal care compared to women receiving standard care. In Fernandez et al. [37] review, studies including women with low or high risk of pregnancy complications and or preterm birth were eligible for inclusion. The review investigated various antenatal care models such as midwife-led model of care, preterm prevention programmes, clinic-based specialised care and standalone intervention. The overall risk of preterm birth was reduced by 16% by implementing alternative care model. However, subgroup meta-analysis including specialist antenatal care studies showed no significant difference when compared with standard antenatal care on reducing preterm birth. These results were derived from combining data from six randomised controlled trials, three of these are included in this review [21, 22, 25].

### Implications of research

There are numerous papers in the literature dealing with interventions to prevent preterm birth, however there is still a gap to identify which interventions are most effective in improving preterm birth maternal and perinatal outcomes [38]. Specialist preterm birth clinics provide a complex package of care and thus, with an agreed standard protocol and guidelines on screening criteria, diagnostic tests and a treatment plan for women attending the clinic. Future studies should include a standardized reporting of the intervention and the relevant outcomes as well as establishing a standardized economic model. More research in screening tests to predict preterm birth is also needed. A well-designed cluster randomisation study would be therefore the preferred design to establish the efficacy of such an intervention, but this approach might be hard to achieve as such clinics are currently a well-established means of providing antenatal care for high risk women in many settings. However, given the heterogeneity of clinics and variations in practice [14, 39], such a study has not yet been undertaken. Women’s well-being, mental health and satisfaction and experience of care provided and that of their partners should be included in the design of future studies. Fathers support and experience of PTB is also in need of further research.

## Conclusion

There is no evidence yet, either in support of or to refute the effect of a preterm prevention clinic in reducing preterm birth. However, this kind of specialist clinic serves the purpose of offering coordinated and individualized antenatal care to women at high risk of preterm labour. Further clarification is necessary on the optimal referral route and a clear and standardized management plan for this service.

## Appendix A

**Table 5:** Medline Search results in March 2015

1	((specialist or specialised or specialized) adj3 clinic?).ti,ab.	2620
2	((specialist or specialised or specialized) adj3 (class or classes)).ti,ab.	124
3	((specialist or specialised or specialized) adj3 meeting?).ti,ab.	60
4	((specialist or specialised or specialized) adj3 service?).ti,ab.	3588
5	1 or 2 or 3 or 4	6279
6	(pregnan* or antenatal or ante-natal or antepartum or ante-partum or prenatal or pre-natal or prepartum or pre-partum or preterm or pre-term).ti,ab,hw.	798,714
7	5 and 6	294
8	((miscarriage? or high risk pregnan* or pregnancy complication? or complicated pregnanc* or pre-eclampsia or eclampsia or gestational diabet* or gestational hypertens* or pregnancy induced hypertens*) adj3 clinic?).ti,ab.	108
9	((miscarriage? or high risk pregnan* or pregnancy complication? or complicated pregnanc* or pre-eclampsia or eclampsia or gestational diabet* or gestational hypertens* or pregnancy induced hypertens*) adj3 service?).ti,ab.	29
10	7 or 8 or 9	425
11	Prenatal Care/og [Organization & Administration]	1081
12	Prenatal Care/and (ambulatory care facilities/or outpatient clinics, hospital/)	282
13	Pregnancy/and (ambulatory care facilities/or outpatient clinics, hospital/)	1067
14	((antenatal or ante-natal or antepartum or ante-partum or prenatal or pre-natal or prepartum or pre-partum or preterm or pre-term) adj3 clinic?).ti,ab.	4113
15	((antenatal or ante-natal or antepartum or ante-partum or prenatal or pre-natal or prepartum or pre-partum or preterm or pre-term) adj3 (class or classes)).ti,ab.	395
16	((antenatal or ante-natal or antepartum or ante-partum or prenatal or pre-natal or prepartum or pre-partum or preterm or pre-term) adj3 meeting?).ti,ab.	17
17	((antenatal or ante-natal or antepartum or ante-partum or prenatal or pre-natal or prepartum or pre-partum or preterm or pre-term) adj3 service?).ti,ab.	1134
18	((antenatal or ante-natal or antepartum or ante-partum or prenatal or pre-natal or prepartum or pre-partum or preterm or pre-term) and clinic?).ti.	565
19	((antenatal or ante-natal or antepartum or ante-partum or prenatal or pre-natal or prepartum or pre-partum or preterm or pre-term) and service?).ti.	300
20	11 or 12 or 13 or 14 or 15 or 16 or 17 or 18 or 19	7461
21	*Pregnancy, High-Risk/	1611
22	*Abortion, Habitual/	3894
23	((recur* or history or habitual) adj3 (miscarriage* or abortion?)).ti,ab.	4898
24	*Hypertension/or *Hypertension, Pregnancy-Induced/	133,497
25	*eclampsia/or *hellp syndrome/or *pre-eclampsia/	19,170
26	(hypertens* or high blood pressure or eclampsia or pre-eclampsia or preeclampsia).ti.	160,268
27	*Diabetes Mellitus, Type 1/or *Diabetes, Gestational/	51,216
28	(diabet* and (gestational or pregnan*)).ti.	8126
29	Placenta Previa/	2183
30	(placenta previa or placenta praevia).ti,ab.	2165
31	Pregnancy Complications/	70,144
32	((high risk* or complicat*) and pregnan*).ti.	6659
33	*HIV Infections/or *HIV Seropositivity/or exp *HIV/	161,063
34	(hiv or hiv1 or hiv2 or human immunodeficiency virus).ti.	162,191
35	exp *Sexually Transmitted Diseases/	226,680
36	21 or 22 or 23 or 24 or 25 or 26 or 27 or 28 or 29 or 30 or 31 or 32 or 33 or 34 or 35	609,992
37	20 and 36	2328

* is a truncation symbol and was used to retrieve terms with a common root within MEDLINE search

## Abbreviations

CASP: Critical Appraisal Skills Programme
EPOC: Cochrane Effective Practice and Organization of Care group (EPOC)
PRISMA: Preferred Reporting Items for Systematic Reviews and Meta-Analyses
PTB: Preterm birth
RCTs: Randomised Controlled Trials

## References

[1] Wold Health Organization (WHO). Preterm birth. 2015. http://www.who.int/mediacentre/factsheets/fs363/en/. Accessed 22 Sept 2015.

[2] Office of National Statistics (ONS). Birth characteristics in England and Wales. 2014. http://www.ons.gov.uk/ons/rel/vsob1/birth-characteristics-in-england-and-wales/2014/stb-birth-characteristics-2014.html. Accessed 22 Sept 2015.

[3] Blencowe H, Vos T, Lee AC (2013). Estimates of neonatal morbidities and disabilities at regional and global levels for 2010: introduction, methods overview, and relevant findings from the Global Burden of Disease study. Pediatr Res.

[4] Liu L, Johnson HL, Cousens S, Perin J, Scott S, Lawn JE, Rudan I, Campbell H, Cibulskis R, Li M, Mathers C, Black RE (2012). Child Health Epidemiology Reference Group of WHO and UNICEF: Global, regional, and national causes of child mortality: an updated systematic analysis for 2010 with time trends since 2000. Lancet.

[5] Raju TN (2006). Epidemiology of late preterm (near-term) births. Clin Perinatol.

[6] Blencowe H, Cousens S, Oestergaard MZ, Chou D, Moller AB, Narwal R, Adler A, Vera Garcia C, Rohde S, Say L, Lawn JE (2012). National, regional, and worldwide estimates of preterm birth rates in the year 2010 with time trends since 1990 for selected countries: a systematic analysis and implications. Lancet.

[7] Webb DA, Coyne JC, Goldenberg RL, Hogan VK, Elo I, Bloch JR, Mathew L, Bennett IM, Dennis EF, Culhane JF (2010). Recruitment and retention of women in a large randomized control trial to reduce repeat preterm births: the Philadelphia Collaborative Preterm Prevention Project. BMC Med Res Methodol.

[8] Jackson RA, Gibson KA, Wu YW, Croughan MS (2004). Perinatal outcomes in singletons following in vitro fertilization: a meta-analysis. Obstet Gynecol.

[9] Gilbert WM, Nesbitt TS, Danielsen B (2003). The cost of prematurity: quantification by gestational age and birth weight. Obstet Gynecol.

[10] Petrou S (2003). Economic consequences of preterm birth and low birthweight. BJOG.

[11] Slattery MM, Morrison JJ (2002). Preterm delivery. Lancet.

[12] Kugler JP, Connell FA, Henley CE (1990). Lack of difference in neonatal mortality between blacks and whites served by the same medical care system. J Fam Pract.

[13] Alexander GR, Baruffi G, Mor JM, Kieffer EC, Hulsey TC (1993). Multiethnic variations in the pregnancy outcomes of military dependents. Am J Public Health.

[14] Lamont RF (2006). Setting up a preterm prevention clinic: a practical guide. BJOG.

[15] Newnham JP, Dickinson JE, Hart RJ, Pennell CE, Arrese CA, Keelan JA (2014). Strategies to Prevent Preterm Birth. Front Immunol.

[16] Whitworth M, Quenby S, Cockerill RO, Dowswell T. Specialised antenatal clinics for women with a pregnancy at high risk of preterm birth (excluding multiple pregnancy) to improve maternal and infant outcomes. Cochrane Database Syst Rev. 2011. doi:10.1002/14651858.CD006760.pub2. 10.1002/14651858.CD006760.pub2 PMC4084921 21901705

[17] Moher D, Liberati A, Tetzlaff J, Altman DG, The PRISMA Group (2009). Preferred Reporting Items for Systematic Reviews and Meta-Analyses: The PRISMA Statement. PLoS Med.

[18] Higgins JPT, Green S (editors). Cochrane Handbook for Systematic Reviews of Interventions Version 5.0.0 [updated March 2011]. The Cochrane Collaboration. 2011. http://handbook.cochrane.org/. Accessed 27 Jan 2017.

[19] Effective Practice and Organisation of Care (EPOC): Suggested risk of bias criteria for EPOC reviews. EPOC Resources for review authors. Oslo: Norwegian Knowledge Centre for the Health Services. 2015. http://epoc.cochrane.org/epoc-specific-resources-review-authors. Accessed 8 Jan 2015.

[20] Critical Appraisal Skills Programme (CASP): CASP Qualitative Checklist. http://www.casp-uk.net/#!casp-tools-checklists/c18f8. Accessed 10 Jan 2015.

[21] Iams JD, Johnson FF. Effect of a preterm birth prevention program on the diagnosis and treatment of preterm labor in high risk patients. In: Proceedings of 9th Annual Meeting of the Society of Perinatal Obstetricians. New Orleans, Louisiana, USA; Feb 1-4. Am J Obstet Gynecol. 1989;161:387.

[22] Main DM, Richardson DK, Hadley CB, Gabbe SG (1989). Controlled trial of a preterm labor detection program: efficacy and costs. Obstet Gynecol.

[23] Mueller-Heubach E, Reddick D, Barnett B, Bente R (1989). Preterm birth prevention: evaluation of a prospective controlled randomized trial. Am J Obstet Gynecol..

[24] Goldenberg RL, Davis RO, Copper RL, Corliss DK, Andrews JB, Carpenter AH (1990). The Alabama Preterm Birth Prevention Project. Obstet Gynecol.

[25] Hobel CJ, Ross MG, Bemis RL, Bragonier JR, Nessim S, Sandhu M (1994). The West Los Angeles preterm birth prevention project: I. program impact on high-risk women. Am J Obstet Gynecol.

[26] Herron MA, Katz M, Creasy RK (1982). Evaluation of a preterm birth prevention program: preliminary report. Obstet Gynecol.

[27] Manuck TA, Henry E, Gibson J, Varner MW, Porter FT, Jackson GM, Esplin MS (2011). Pregnancy outcomes in a recurrent preterm birth prevention clinic. Am J Obstet Gynecol.

[28] Karkhanis P, Patni S, Gargeswari S (2012). Performance of the preterm prevention clinic at heart of England NHS trust. Int J Gynaecol Obstet.

[29] Burul G, James CP, Forya F, Casagrandi D, Burul G, James CP, Forya F, Casagrandi D (2014). Does specialist antenatal care for women at risk of preterm birth affect patient selection, rate and outcomes of cervical cerclage?. Arch Dis Fetal Neonatal Ed.

[30] Cohen A, Kindinger L, Clifford K, Bennett P, Teoh TG (2014). Who is most at risk: a preterm surveillance clinic audit. BJOG.

[31] O’Brien ET, Quenby S, Lavender T (2010). Women’s views of high risk pregnancy under threat of preterm birth. Sex Reprod Healthc.

[32] Creasy PK, Gummer BA, Liggins GC (1980). System predicting spontaneous preterm birth. Obstet Gynecol.

[33] Ross MG, Sandhu M, Bemis R, Nessim S, Bragonier JR, Hobel C (1994). The West Los Angeles preterm birth prevention project: II. cost-effectiveness analysis of high-risk pregnancy interventions. Obstet Gynecol.

[34] Esplin MS, O’Brien E, Fraser A, Kerber RA, Clark E, Simonsen SE (2008). Estimating recurrence of spontaneous preterm delivery. Obstet Gynecol.

[35] MacKinnon K, McIntyre M (2006). From Braxton Hicks to preterm labour: The constitution of risk in pregnancy. Can J Nurs Res.

[36] Heaman M, Sprague A, Stewart P (2001). Reducing the preterm birth rate: a population health strategy. J Obstet Gynecol Neonatal Nurs.

[37] Fernandez Turienzo C, Sandall J, Peacock JL (2016). Models of ntenatal care to reduce and prevent preterm birth: a systematic review and meta-analysis. BMJ Open.

[38] James Lind Alliance Priority setting partnership (JLA). Preterm Birth Top 10. 2017. http://www.jla.nihr.ac.uk/priority-setting-partnerships/preterm-birth/top-10-priorities/. Accessed 27 Jan 2017.

[39] Sharp AN, Alfirevic Z (2014). Provision and practice of specialist preterm labour clinics: a UK survey of practice. BJOG.

